# New Appliances and Things Medical

**Published:** 1897-03-13

**Authors:** 


### NEW APPLIANCES AND THINGS MEDICAL.

[We shall be glad to receive, at our Office, 28 & 29, Southampton Street, Strand, London, W.C., from the manufacturers, specimens of all new preparations and appliances which may be brought out from time to time.]

### KEROS: A NEW FLUID BEEF.

(Bouillon Fleet, Limited, Warner Road, Camberwell, S.E.)

We have received a bottle of this new beef preparation. Our examination of it shows that it is one of the most concentrated on the market. It contains an astonishingly high percentage of albuminous and fibrinous principles, and, as far as nutritive qualities are concerned, it has as high a value as the meat from which it is prepared, but, in addition, has the valuabl[illegible]o[illegible]erties of being readily digested and absorbed. The seasoning is very pleasant, and in all respects it deserves to rank very high among invalid specialities.

### SOLIDIFIED SOUP SQUARES.

(E. Lazenby and Son, 18, Trinity Street, London, E.C.)

We have received samples of these well-known and useful soups, and after making careful trial of them we feel completely justified in recommending them to the notice of the profession as being particularly valuable for invalid use. In small households, or in those in which the facilities for cooking are indifferent, they will be found to have their most useful application. Invalids soon tire of domestic and ready prepared beef teas, and will welcome as a most beneficial change some of the clear soups prepared by the above firm. In addition, many of the thick varieties may be safely indulged in by invalids or convalescents under medical supervision; and, as well as being particularly agreeable to the palate, they possess nutritive value of a high grade.

### "PURO" TEA.

We have had a sample of "Puro" tea submitted to our notice, and the result of our investigation leads us to the conclusion that in this decidedly palatable tea will be found the solution of the problem as to what we may recommend certain dyspeptics to drink instead of ordinary tea. It appears to contain about half the average amount of free tannic acid, and thus, while in no way deprived of its palatable properties, the injurious action which so commonly follows the use of tea is largely discounted. Ordinary tea is too often the "bête noir" of a large class of dyspeptics, for not only does it interfere with the processes of a weak digestion, but it may aggravate, or in some cases be the main factor in setting up dyspepsia. In some cases there may be little difficulty in persuading the patient to discontinue tea drinking altogether, but in a large proportion the prohibition of tea involves grave self-denial. We therefore welcome the novel and unique preparation of tea known as "Puro."

